# Linking forest management to moose population trends: The role of the nutritional landscape

**DOI:** 10.1371/journal.pone.0219128

**Published:** 2019-07-16

**Authors:** Thomas V. Schrempp, Janet L. Rachlow, Timothy R. Johnson, Lisa A. Shipley, Ryan A. Long, Jocelyn L. Aycrigg, Mark A. Hurley

**Affiliations:** 1 Department of Fish and Wildlife Sciences, University of Idaho, Moscow, Idaho, United States of America; 2 Idaho Department of Fish and Game, Lewiston, Idaho, United States of America; 3 Department of Statistical Science, University of Idaho, Moscow, Idaho, United States of America; 4 School of the Environment, Washington State University, Pullman, Washington, United States of America; 5 Idaho Department of Fish and Game, Boise, Idaho, United States of America; Cornell University, UNITED STATES

## Abstract

Forested lands in the western USA have undergone changes in management and condition that are resulting in a shift towards climax vegetation. These changes can influence the quality and quantity of forage for herbivores that rely on early-seral plants. To evaluate how management of forested landscapes might affect nutrition for Shiras moose (*A*. *a*. *shirasi*) at large spatial scales, we focused on shrubs and evaluated summer diet composition, forage availability, and forage quality across 21 population management units encompassing >36,000 km^2^ in northern Idaho, USA. We identified 17 shrub species in the diets of moose, 11 of which comprised the bulk of the diets. These forage shrubs varied markedly in both energy (mean digestible energy for leaves ranged from 9.62 to 12.89 kJ/g) and protein (mean digestible protein for leaves ranged from 1.73 to 7.90%). By adapting established field sampling methods and integrating recent advances in remote sensing analyses in a modeling framework, we predicted approximations of current and past (i.e., 1984) quantities of forage shrubs across northern Idaho. We also created a qualitative index of population trend for moose across population management units using harvest data. Predicted quantities of forage shrubs varied widely across the study area with generally higher values at more northern latitudes. The quantity of forage shrubs was estimated to have declined over the past 30 years in about half of the population management units, with the greatest declines predicted for high-energy forage species. The population trend index was correlated with the percent change in availability of moderate-energy forage shrubs, indicating that availability of forage shrubs and change in availability over time might be affecting population dynamics for moose in northern Idaho. Our study highlights the importance of assessing how changes in forest management across broad spatiotemporal extents could affect wildlife and their habitats.

## Introduction

Forested lands in the western USA have undergone marked shifts in management and condition over the past century, and understanding how these changes have influenced wildlife is critical for long-term maintenance of productive wildlife populations and ecological communities. Timber harvest has a long history in western forests, and indeed, the Organic Administrative Act of 1897 directed land managers to “furnish a continuous supply of timber” [1]. However, passage of the Multiple Use-Sustained Yield Act in 1960 signaled a growing recognition of non-utilitarian values for national forest lands [2] and a subsequent decline in timber harvest [3]. Fire management policies for national forests also have changed in the past century; fire suppression in the northern Rocky Mountains became effective in the 1930s, reducing the frequency and intensity of wildfires on national forests [4]. Fire is important for maintaining early-seral vegetation communities [5–6], and long-term fire suppression has resulted in a shift towards climax vegetation [7]. Because of their influence on successional processes, fire suppression and the reduction of timber harvest have the potential to reduce quality and quantity of forage for herbivores that rely on early-seral plants, thereby imposing nutritional limitations on population productivity.

Management practices that alter forest succession can affect ungulates by changing forage availability [8–9] and quality [10–11], and by influencing patterns of habitat use, selection and movements [12–13]. Such changes can lead to nutritional limitations and have pronounced effects on individual fitness, and ultimately, population dynamics [14]. Evidence of inadequate nutrition limiting mass gain by adult or juvenile ungulates has been reported for elk (*Cervus canadensis*; [15]), roe deer (*Capreolus caperolus*; [16]), mule deer (*Odocoileus hemionus*; [17]), and moose (*Alces* alces; [18]). Similarly, pregnancy rates have been linked to nutrition and body condition [19]. In addition, body mass and condition of neonates, juveniles, and adults has been correlated with survival for caribou (*Rangifer tarandus*; [20]), elk [21–22], mule deer [23]), and moose [24].

Despite long-standing emphasis on winter as a nutritional bottleneck for ungulates, a growing body of evidence indicates that summer nutrition is equally, if not more, important in temperate ecosystems. If summer nutrition is limited, females can exhibit delayed age at first reproduction and reproductive pauses [15, 25]. Moreover, in addition to needing adequate nutrition to support pregnancy and lactation, females must recoup body mass lost over the previous winter in preparation for the coming winter [15, 26]. Failure to recover sufficient fat reserves during summer can predispose individuals to mortality from diverse proximate causes, such as predation [27] or parasites [28–29]. Indeed, the role of summer nutrition in regulating reproduction and survival has been documented for numerous ungulates [19, 30] including moose via twinning rates [31], recruitment [32], and survival [27].

Moose populations are declining in much of North America, including parts of the western, upper-midwestern and eastern USA and southcentral and western Canada. Population declines in Shiras moose (*A*. *a*. *shirasi*), the subspecies that inhabits the Northern Rocky Mountains, have been documented in Montana [33], Wyoming [34], and parts of Idaho; however, other populations in Idaho and Washington have increased [35]. Many potential correlates of moose population changes have been examined, however, the factors affecting survival and reproduction, and ultimately population growth or decline, remain poorly understood for Shiras moose [19].

We developed an approach for synthesizing diverse data on diet, forage availability, and forage quality across broad spatial extents to understand the potential for nutritional regulation of Shiras moose populations in managed forest landscapes. We focused on woody shrubs consumed by moose in our study region. We applied this approach across an area of 36,654 km^2^ in northern Idaho where forest management practices have resulted in a high proportion of late-seral forest. Our objectives included 1) evaluating composition of woody shrubs in the diet, 2) assessing nutritional quality of forage shrubs consumed by moose, 3) estimating availability and quality of forage shrubs across the landscape, 4) estimating changes in forage conditions over several decades, and 5) interpreting results in the context of a qualitative population index to evaluate how landscape nutrition might be linked to moose population trends. We predicted that individuals would consume forage species that were both highly available and highly to moderately digestible because moose are large, selective browsers [36]. We also expected that an index of population change would be positively correlated with both quantity of forage shrubs and changes in availability of those shrubs over time. Our study illustrates an approach for evaluating nutritional consequences of land management over broad spatial extents that could be applied in other forested ecosystems. Moreover, our application of this approach provides a foundation for evaluating whether nutritional limitation might be affecting moose population dynamics in northern Idaho and elsewhere.

## Methods

### Study area

**Fig 1:**
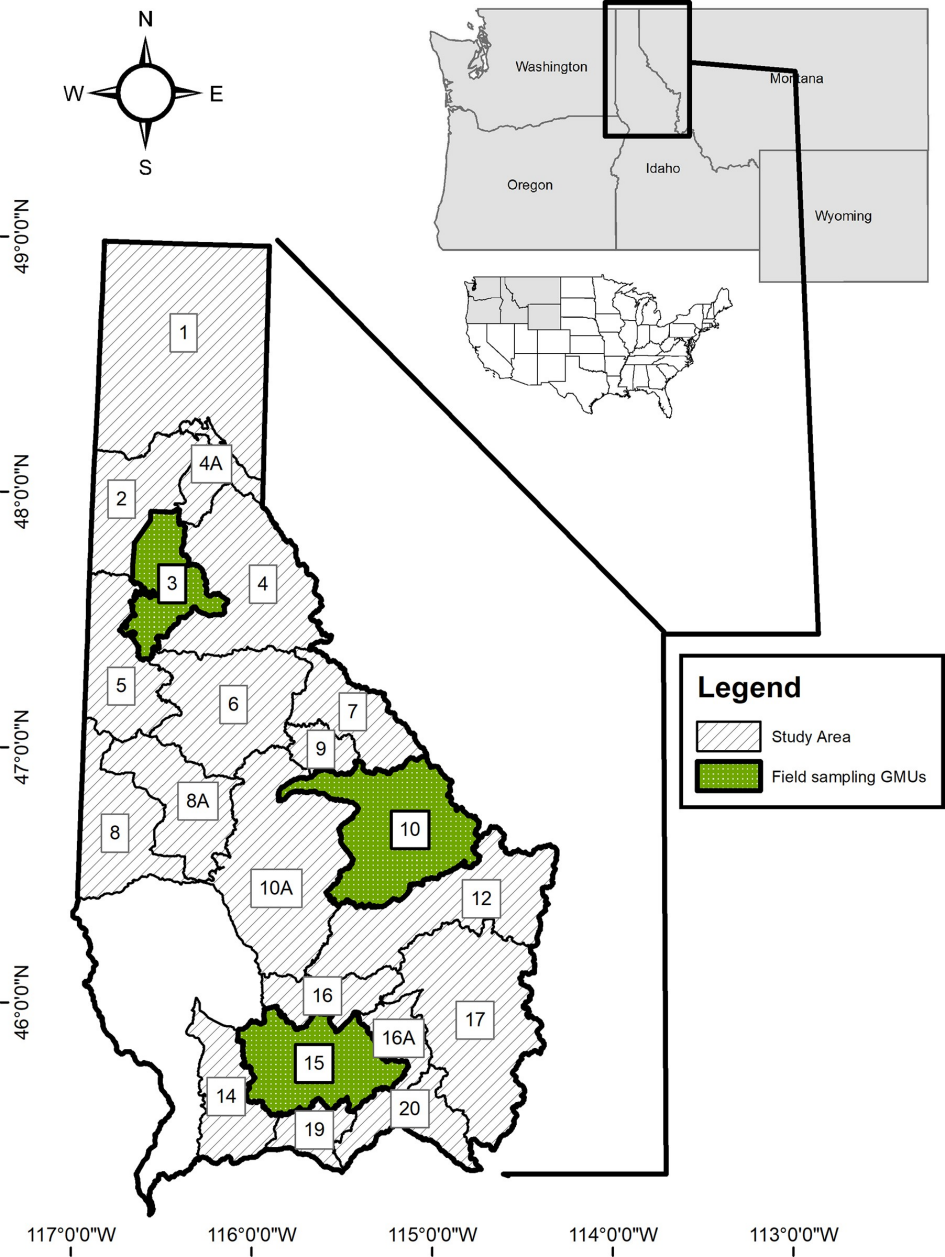
Study area map. Location of the study area and Game Management Units (GMUs) in northern Idaho, USA.

We conducted this research in northern Idaho, USA (Fig 1), across 21 game management units (GMUs; areas within which wildlife populations are managed) that are delineated based on geographic features, vegetation communities, and land use. The study area (>36,000 km^2^) was generally mountainous and dominated by coniferous forests with limited areas of riparian or meadow habitats with aquatic vegetation. Annual precipitation was higher in the northeastern part of the study area, whereas average summer temperatures were higher in the southwestern portion (S1 Table). Land ownership consisted predominately of forests managed by the U.S. Forest Service, but also included Idaho state endowment lands, corporate timber lands, and private property (S1 Table). The following trees and shrubs occurred across the study region: western hemlock (*Tsuga heterophylla*), grand fir (*Abies grandis*), Douglas-fir (*Pseudotsuga menziesii*), subalpine fir (*Abies lasiocarpa*), western red cedar (*Thuja plicata*), western white pine (*Pinus monticola*), western larch (*Laryx occidentalis*), ponderosa pine (*Pinus ponderosa*), lodgepole pine (*Pinus contorta*), Engelmann spruce (*Picea engelmannii*), Rocky Mountain maple (*Acer glabrum*), scouler willow (*Salix scouleriana*), redstem ceanothus (*Ceanothus sanguineus*), evergreen ceanothus (*Ceanothus velutinus*), rusty menziesia (*Menziesia ferruginea*), huckleberry species (*Vaccinium spp*.), cherry species (*Prunus spp*.), western thimbleberry (*Rubus parviflorus*), common snowberry (*Symphoricarpos albus*), alder species (*Alnus spp*.), mallow ninebark (*Physocarpus malvaceus*), and oceanspray (*Holodiscus discolor*).

### Diet composition

Field sampling for diet composition and forage analyses occurred simultaneously during 2015–2016 (sampling design detailed below in ‘Field sampling for forage quantity’). We located fresh fecal samples opportunistically while conducting vegetation transects during July—September and randomly collected 6 to 8 pellets from each pellet group to estimate summer diet composition via microhistological analysis. Samples were collected within 2 GMUs selected to represent the broader study area. Freshness was evaluated visually based on the surface color and texture, and on the interior color and moisture; fresh pellets were dark and smooth externally with greenish interiors and moist interiors. Yearling fecal pellet groups weigh about 75% more than calf fecal pellet groups [37], and so we excluded fecal samples from calves based on fecal group and pellet size, and we avoided collecting multiple samples from the same area (i.e., within approximately 1 km) to minimize repeat sampling of the same individuals. Sex for each sample was unknown, but we assumed that diets did not differ markedly between sexes [38]. Samples were analyzed by the Micro Composition Laboratory in Boulder, Colorado, USA. Each sample was viewed 60 times (20 views on 3 slides), and forage shrubs were identified to the species level when possible. Using the equation for digestible dry matter from [39], we corrected dietary proportions for digestibility (see methods for “Forage relative quality” for additional information). After correcting for digestibility, shrub species that comprised <3% of the samples averaged across GMUs were excluded from further analyses because these forage species were likely consumed incidentally and contributed the least to the overall diet. We focused on shrubs based on prior studies assessing moose diets in our area [40] and because grasses and forbs senesce and decline in quality to the point that we did not expect them to be major components of the diet during summer.

### Field sampling for forage quantity

**Fig 2:**
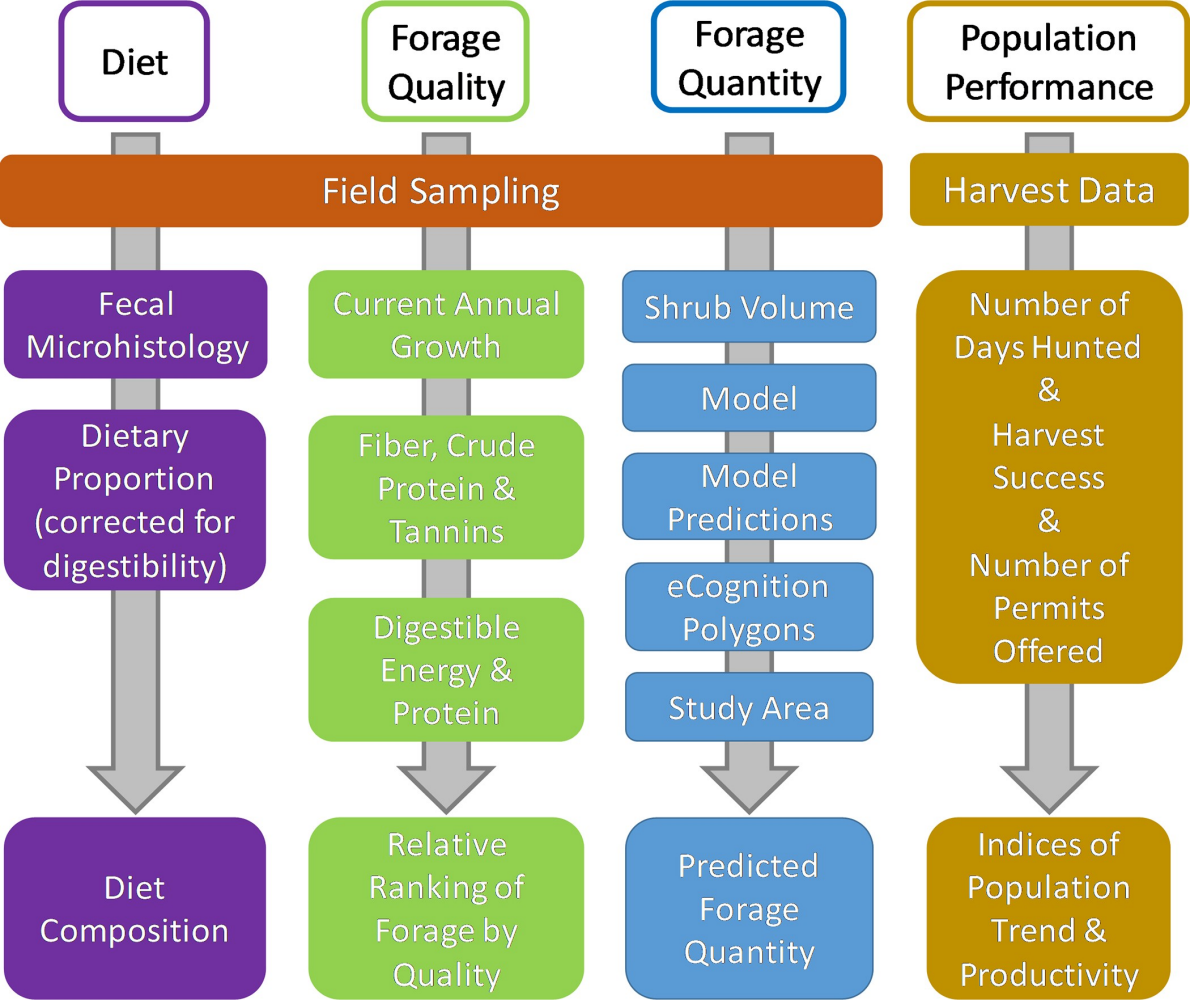
Data processing steps. Data generation and processing steps for each study objective for evaluating moose forage and nutrition across 36,654 km^2^ in northern Idaho, USA.

We conducted field sampling to collect data for modeling forage quantity and to collect samples for analyses of forage quality (Fig 2) during summer (July to September) of 2015 and 2016 in 3 GMUs (GMU3, GMU10, and GMU15; Fig 1) that spanned the range of forest types, successional stages, and land uses representative of the broader study area. Field sampling was conducted in 2 stages. First, we employed a design-based sampling methodology to assess general trends in species composition and associated growing conditions. Second, we adapted our sampling to target moose forage species in a spatially balanced manner [41] that facilitated subsequent model-based prediction of variation in forage quantity across space and time.

Stage 1 was accomplished in 2015 using a stratified random sampling design with allocation proportional to stratum area. Strata were created using potential natural vegetation (PNV) types [42–43], grouped by the dominant tree species present at climax, and LANDFIRE [44] canopy cover data (binned into intervals of 0 to < 30%, 30 to < 60%, and ≥ 60%). Potential natural vegetation represents the relatively stable end-product of succession that is in equilibrium with its environment, and thus is a biologically useful index of factors (e.g., elevation, aspect, precipitation, soils, etc.) that influence plant ecology [42]. Riparian areas along rivers and streams comprised the final stratum for a total of 8 strata. Within strata, we randomly distributed field sampling locations across the range of cumulative solar radiation received in each GMU from May 1 to August 31 (estimated using the ‘area solar radiation’ tool in ArcMap 10.3; ArcGIS 10, ESRI, Redlands, CA). The ‘area solar radiation’ tool estimates incoming solar radiation for pixels within a digital elevation model (DEM) while accounting for variation in elevation, slope, aspect, surrounding topography, latitude, time of year, and time of day [45]. We chose to stratify across solar radiation values because the calculation of solar radiation incorporates the effect of multiple factors on how much sunlight a location receives, which in turn influences plant ecology through photosynthesis, soil temperature, and soil moisture [45]. Solar radiation values within each stratum were grouped into high, medium, and low bins for stratifying, and field sampling locations were allocated equally among bins.

At each field sampling location, we measured vegetation along line transects (n = 235) that were 180 m in length with random starting points and directions. We estimated shrub crown volume as an index to biomass of current annual growth (CAG) of shrubs. Each transect included 4 circular plots with a 2-m radius (12.56 m^2^) placed every 60 m, within which we took 3 measurements of each shrub to estimate crown volume: height, widest canopy diameter, and canopy diameter perpendicular to the widest diameter. We also recorded if CAG was browsed by an ungulate as indicated by removal of stems or leaves at heights of >1m above the ground. When the density of a shrub species was high (≥ 20 shrubs per plot), the average crown volume of a representative subsample of shrubs (n = 5–14) was multiplied by the total count. We also recorded canopy cover using a densiometer. To evaluate the relationship between linear shrub measurements and biomass of CAG and our ability to use shrub volume as an estimate of forage quantity, we collected and oven-dried CAG for 34 willow shrubs, which are known to be important for moose. Crown volume was strongly correlated with biomass of CAG (*R*^2^ = 0.74, *n* = 34), and consequently, we used volume of forage shrubs to model forage quantity across the landscape. Although we did not repeat this assessment for each shrub species, we assumed that a similar positive relationship existed between shrub size and biomass based on other published studies reporting similar relationships [46–47].

The second stage of field sampling was accomplished in 2016, when we adapted our sampling design to target key forage species based on preliminary diet analyses of fecal samples and shrub data collected during 2015. To improve sampling efficiency, we shortened transect length to 90 m and placed plots at 30-m intervals. In addition, we only recorded the shrub diameter that was perpendicular to the widest diameter because this single measurement was strongly correlated with shrub volume across all forage species (mean *R*^2^ = 0.92, range 0.86 to 0.98, n = 18–444 per forage species). The closed-canopy strata were larger than open-canopy strata, and therefore, closed-canopy sites were sampled more intensely in 2015. However, most forage species identified in the preliminary analysis of diet composition were associated with open-canopy forests. To target these forage species, we focused sampling during 2016 on polygons of open forests (< 50% closure) identified in LANDFIRE and attributed with environmental data including percent shrub cover, fire history (i.e., burned or not since 1985), elevation, potential natural vegetation, and aspect. We chose a spatially balanced sampling (SBS) design and used the “Create Spatially Balanced Points” tool in ArcGIS 10.3 to create spatially balanced starting points with random transect bearings. This tool uses the Reverse Randomized Quadrant-Recursive Raster (RRQRR) developed by Theobald et al. 2007 [41] to maximize spatial independence. Natural resource data typically exhibit spatial autocorrelation, and SBS designs increase the information yielded per sampling unit by maximizing spatial independence among samples and distributing sampling effort across the study area [41]. A total of 386 transects were completed in 2016 for a combined total of 621 transects during the study.

### Predicted current and past forage quantity

We constructed models for predicting presence and volume of forage shrubs across the landscape by first creating 10-m diameter buffers around each transect and attributing them with environmental covariates with the potential to influence shrub growth (S2 Table). Variables included solar radiation (described above), terrain features (e.g., elevation, aspect), soil properties, climatic parameters associated with precipitation and temperature, forest closure, and time since fire (S2 Table). We used regularized “lasso” regression [48] to model shrub species presence and volume per meter-squared for each forage species. Presence was modeled using regularized logistic regression with the R package ‘glmnet’ [49], and volume per meter-squared was modeled using regularized gamma regression with the R package ‘gamlr’ [50]. Regularized models append a penalty term to the likelihood function that helps avoid excessive overfitting caused by a large number of potential covariates relative to the number of observations by “shrinking” the regression coefficients towards zero. Some of the coefficients can be set to zero in this process, effectively dropping them from the model. By evaluating the predictive accuracy of the regularized model using cross-validation at different levels of regularization, a model can be selected that attempts to produce the best possible predictions given the available data to estimate the model. As a byproduct of this process, prediction error due to near collinearities among the covariates also can be reduced. A K-fold cross-validation was used to set the regularization by maximizing the estimated area under the receiver-operating curve (ROC) for the presence models, and minimizing the estimated mean squared error (MSE) of prediction for the volume models. The cross-validation was repeated 30 times to reduce variability in the results due to random allocation of the observations to sub-samples used for cross-validation. The R package ‘PresenceAbsence’ [51] was used to optimize presence-absence thresholds and estimate predictive accuracy and Cohen’s Kappa [52]. Probability thresholds for each forage species were selected so that specificity equaled sensitivity. We extrapolated our models across the landscape by identifying homogenous areas (i.e., polygons) throughout our study area using object-oriented analysis (eCognition software; Trimble Inc. Westminster, CO) to segment 1-m aerial imagery based on spectral values of red, green, blue, and near infrared [53]. These polygons were attributed with covariate data (S2 Table), and shrub presence and volume were predicted for each forage species using the ‘glmnet’ and ‘gamlr’ packages in Program R.

To evaluate change in availability of moose forage over time, we modeled forage quantity across northern Idaho in 2016 using remotely sensed imagery and compared current values to estimates based on forest conditions 3 decades earlier (using 1984 Landsat imagery, which was the earliest imagery available that was cloud free). Forage models to predict current forage quantity used tree canopy cover from the 2011 National Land Cover Database [54] because more recent disturbances would likely over-estimate current forage quantity because of the delay in recruitment and growth of shrubs post-disturbance. We estimated historic canopy cover in 1984 using changes in reflectance of Landsat imagery between 1984 and 2016 (S1 Text). Values for the covariate time since fire also were corrected for conditions in 1984. We then used model parameters that were estimated using 2015–2016 data to generate forage quantity predictions for 1984 based on estimated canopy cover and time since fire covariate values for that year.

### Forage relative quality

Forage shrub species were ranked based on their relative digestible energy (DE) and digestible protein (DP) values. Samples were collected while conducting field work to model forage quantity (sampling design described previously), and hence samples were stratified by the biological variables described above. We collected 220 samples of current annual growth (CAG) throughout the 2015–16 field seasons and stored them at -20° C until they were freeze-dried and ground in a cyclone mill with a 1.0-mm screen. At each sampling location, clippings of CAG were taken at multiple browsing heights from multiple shrubs and then frozen by the end of each field day. We separated leaves and stems for analyses, and composited ground samples by species and plant part. Samples were composited because we were not evaluating spatiotemporal variation in quality, but simply estimating an overall ranking of relative quality to test our hypothesis that changes in forage quantity, especially changes in high and moderate quality forage, are influencing moose populations. To estimate DE (kJ/g) and DP (g per 100 g of forage), we first measured cell wall constituents (%) using sequential fiber analyses [55] and crude protein (%) via combustion [55] at a commercial lab (Dairy One, Ithaca, NY). Fiber analyses were modified for tanniferous browse by including sodium sulfite [56]. Tannin protein-precipitating capacity (mg/mg forage dry matter, [57] was assessed at the Wildlife Habitat and Nutrition Lab (Washington State University, Pullman, WA). Digestible protein (DP) and digestible dry matter (DDM) for each forage species were estimated with equations from [58] and [39], respectively. We used published mean gross energy (GE, kJ/g) values for leaf and stem material from [59] to calculate digestible energy (DE = GE × DDM) because GE for leaf and stem material vary little among species [59]. We categorized shrub species as high, moderate, or low energy based on their estimated DE (S1 Text). We also ranked shrubs according to whether their protein content was sufficient to offset daily metabolic fecal nitrogen and endogenous urine nitrogen losses. The estimated DP needed to offset the loss was 4.30 g/100g dry matter (S1 Text). Shrubs with DP values <4.3 g/100g were categorized as low in protein. Values between 4.3 and 6.5 g/100g were considered moderate, and shrubs with DP values >6.5 g/100g dry matter were categorized as high.

### Moose population index

To estimate a qualitative index of population trend across northern Idaho from 1984 to 2016, we summarized harvest data collected by the Idaho Department of Fish and Game (IDFG). Because data necessary to estimate a kill-per-unit-effort [60–61] were not available in all years or units, we used available data to assign integer values to each GMU based on changes through time in harvest success, number of days hunted, and the number of permits offered (S3 Table). We assigned these qualitative values over the entire 30-year period to minimize potential biases related to the process by which harvest seasons are set. In the absence of population estimates or demographic parameters such as survival or reproduction, wildlife managers typically use harvest success and hunter effort to adjust permit numbers for both males and females. Consequently, permit levels become a reflection of a manager’s perception about how hunter effort and success relate to population status. In addition, the boundaries of moose hunt areas have remained generally stable over time, thus trends in harvest data within a GMU should reflect changes in the moose population for that GMU over the relatively long 30-year time period. Although we interpret these data only qualitatively, the combination of declining harvest success, increasing number of days hunted, and declining permit numbers have indicated population declines in other moose populations [33]. Assigned values were summed across the 3 data sources for each GMU to produce an overall index value, which ranged from -5 to 5, with negative values indicating population declines and positive values indicating population increases. We evaluated Pearson correlations between the population trend index and metrics of forage quantity and quality.

## Results

### Diet composition

When averaged across samples and GMUs, shrubs comprised 59% of moose diets, followed by grasses (18%), conifers (15%), and forbs (7%). Shrub species composition and the mean contribution of shrubs did not differ significantly between sampling areas (27 fecal samples collected in GMU3 and 16 in GMU15) based on bootstrapped 95% confidence intervals. A species accumulation curve showed that all forage species were detected with 10 or more fecal samples, indicating that our sample size was adequate for detecting primary forage species. Mean dietary proportions, which are the proportion of each shrub species averaged across all individual diets, exceeded 3% for eleven of the 17 forage shrubs identified (S4 Table). These more heavily consumed shrubs occurred in ≥12% of the individual diets, and three forage shrubs (willow spp., bitter cherry, and mallow ninebark) occurred in ≥60% of the individual diets (Table 1). Variability in the individual dietary proportions of these shrubs was high; snowberry had the smallest range (3–12%) and ceanothus spp. had the largest range (3–82%), followed by salix spp. (3–53%). Although ceanothus spp. were documented in only 1/3 of the individual diets, within those diets their contribution averaged 24%. Similarly, Pacific yew, a conifer that we treated as a shrub due to its growth form, occurred in 12% of the individual diets, but within those diets its contribution averaged 24%. Western red cedar and western hemlock, which occurred in 9% and 7% of the individual diets, respectively, were the only conifer species with a mean dietary proportion >3%. The percent of shrubs with evidence of browsing by ungulates varied across shrub species (Table 1), with redstem ceanothus exhibiting the heaviest browsing (70% of sampled shrubs). Evergreen ceanothus was browsed substantially less (only 30% of shrubs were browsed), and thimbleberry was browsed the least (15%).

**Table 1:** Forage shrubs in the diets of moose based on microhistological analyses of 43 fecal samples (diets) collected in in northern Idaho, USA. Reported are the percent occurrence of shrubs in the diets, proportion of the diet composed of each shrub species, percent of sampled plants with evidence of ungulate browsing, digestible energy, and digestible protein.

	Percent Occurrence in Diets	Mean Dietary Proportion	Percent with Browsing	Digestible energy (kJ/g) leaf (stem)	Digestible protein (g/100g) leaf (stem)
Willow spp.	88%	14%	58%	9.6 (7.7)	5.44 (0.39)
Mallow ninebark	63%	17%	24%	10.5 (6.0)	1.73 (0.29)
**Bitter cherry**	60%	13%	41%	10.9 (6.7)	**7.39 (0.58)**
**Alder-birch spp.**	49%	14%	32%	10.0 (9.5)	**7.37 (3.72)**
**Redstem ceanothus**	33%^a^	24%^a^	70%	**11.6 (6.4)**	**7.90 (1.08)**
**Evergreen ceanothus**	33%^a^	24%^a^	30%	**11.6 (8.2)**	**6.69 (1.78)**
Honeysuckle	33%	10%	29%	10.3 (5.0)	4.21 (0.96)
**Redosier dogwood**	19%	12%	60%	**12.1 (8.5)**	5.79 (1.35)
Common snowberry	16%	7%	29%	10.4 (5.2)	5.65 (0.77)
Huckleberry spp.	16%	8%	20%	10.3 (7.5)	4.02 (3.62)
Thimbleberry^b^	14%	6%	15%	11.0 (6.8)	6.37 (-0.18)
**Pacific yew**^c^	12%	24%	38%	**12.9 (NA)**	2.23 (NA)

Mean digestible energy (DE) and digestible protein (DP) on a dry matter basis for leaves and stems of shrubs consumed by moose in northern Idaho, USA. Ungulate browsing could include deer and elk browsing in addition to moose. Bold font indicates high-energy (leaf DE >11.3 kJ/g) or high-protein (leaf DP >6.5g/100g) forage species.

^a^Ceanothus spp. could not be differentiated in the fecal samples.

^b^The negative protein value for thimbleberry stem indicates insufficient protein to offset metabolic loss.

^c^Leaves and stems were analyzed together because field observations suggested moose do not strip leaves from conifers (Pacific yew) as they do deciduous shrubs.

### Predicted current and past forage quantity

Environmental parameters successfully predicted presence of forage shrubs across the landscape. Forage presence models (*n* = 12) constructed using 10-m diameter buffers around field transects (*n* = 621 transects) that were attributed with covariates (S2 Table) had a mean percent correctly classified (PCC) of 75.3% (range = 70.1% to 80.7%), a mean AUC value of 0.774 (range = 0.672 to 0.853), and a mean kappa value of 0.340 (range = 0.130 to 0.532; Table 2). Covariates selected by the cross-validated lasso regression varied among forage species as did mean coefficient values (S5 Table). Although covariates (S2 Table) were informative for predicting shrub presence across the landscape, they did not explain variation in shrub volume. The lasso cross-validation for shrub volume models resulted in intercept-only models for each shrub species, indicating that covariates lacked predictive power. Therefore, we used empirical estimates of the mean volume (cm^3^/m^2^) of each forage species (Table 2) in concert with predicted presence to estimate relative forage quantity across the landscape. For our prediction of forage shrub presence, our object-oriented analysis identified approximately 12.3 million polygons, with a mean area of 2,980 m^2^ (SD = 5,300 m^2^). Within each GMU, the total area (m^2^) of all polygons in which a forage species was predicted to occur was multiplied by the mean shrub volume (cm^3^/m^2^) for that species to estimate the total volume within occupied polygons. This value was divided by the total area of the GMU to estimate the average volume per m^2^ across the GMU for use in relative comparisons across space and time.

**Table 2:** Mean ($\bar{x}$) and standard deviation (SD) of model fit statistics for models predicting presence of forage shrubs consumed by moose in northern Idaho, USA.

Shrub	AUC $\bar{x}$ (SD)	Kappa $\bar{x}$ (SD)	PCC $\bar{x}$ (SD)	Shrub Volume (cm^3^/m^2^) (SD)
Willow spp.	0.726 (0.022)	0.348 (0.037)	0.703 (0.018)	167,194 (263,161)
Mallow ninebark	0.853 (0.021)	0.492 (0.045)	0.790 (0.019)	103,025 (188,553)
Bitter cherry	0.821 (0.021)	0.398 (0.039)	0.766 (0.017)	64,376 (121,279)
Alder-birch spp.	0.774 (0.036)	0.200 (0.043)	0.702 (0.022)	255,030 (266,762)
Redstem ceanothus	0.826 (0.026)	0.335 (0.039)	0.766 (0.017)	80,404 (117,593)
Evergreen ceanothus	0.758 (0.026)	0.251 (0.036)	0.701 (0.018)	105,991 (125,974)
Honeysuckle	0.672 (0.041)	0.256 (0.046)	0.753 (0.021)	7,144 (9,089)
Redosier dogwood	0.776 (0.054)	0.193 (0.048)	0.807 (0.015)	63,559 (104,913)
Common snowberry	0.797 (0.024)	0.476 (0.043)	0.747 (0.021)	4,557 (27,776)
Huckleberry spp.	0.825 (0.019)	0.532 (0.034)	0.767 (0.017)	24,433 (28,217)
Thimbleberry	0.755 (0.020)	0.467 (0.035)	0.736 (0.018)	19,634 (46,772)
Pacific yew	0.703 (0.064)	0.130 (0.038)	0.798 (0.016)	86,058 (217,652)

Area under the curve (AUC) of the receiver operating characteristic, Cohen's Kappa (Kappa), and percent correctly classified (PCC) generated by cross-validation of the lasso regression model repeated 30 times to reduce variability in the results due to random allocation of the observations to sub-samples used for cross-validation. Also reported is the mean shrub volume (cm^3^/m^2^) and standard deviation for each forage shrub.

**Fig 3:**
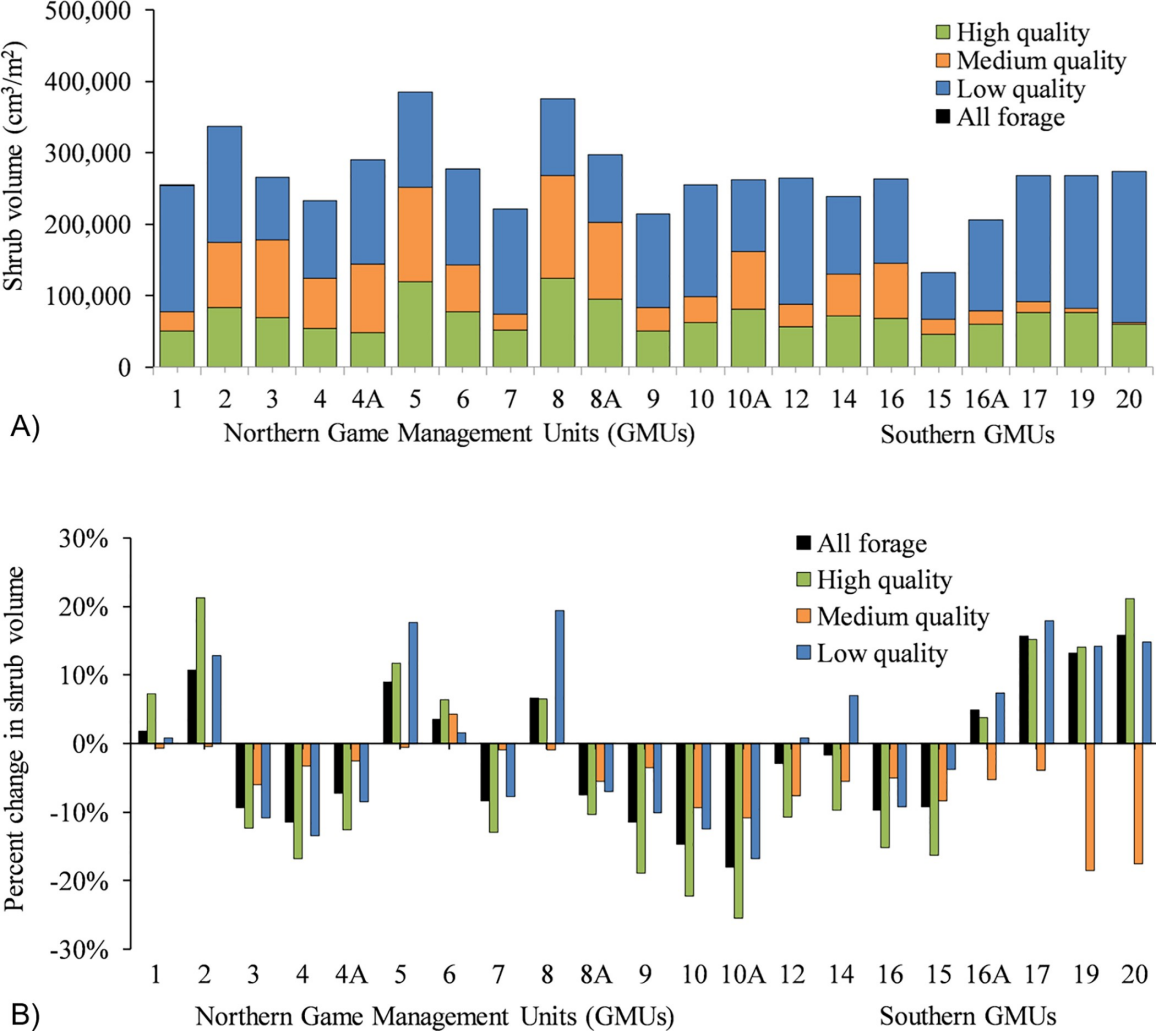
Change in estimated shrub volume. (A) Estimated shrub volume (cm^3^/m^2^) for high, moderate, and low-energy forage shrubs, and (B) percent change from 1984 to 2016 in volume of total forage shrubs, and high-energy and moderate-energy shrubs consumed by moose in northern Idaho, USA, in 21 Game Management Units (GMUs).

Predicted quantities of forage shrubs varied markedly among GMUs (Fig 3A). Northern GMUs associated with western red cedar PNV had greater predicted abundance of forage ($\bar{x}$ ≈ 3.1 × 10^5^ cm^3^/m^2^, range = 2.6 × 10^5^ to 3.9 × 10^5^ cm^3^/m^2^), whereas southern GMUs dominated by grand fir or subalpine fir PNV tended to have relatively less forage ($\bar{x}$ ≈ 2.4 × 10^5^ cm^3^/m^2^, range = 1.3 × 10^5^ to 2.7 × 10^5^ cm^3^/m^2^).

The quantity of moose forage was estimated to have declined over the past 30 years in about half of the GMUs, with the greatest declines predicted for high-energy forage species (Fig 3B). Total forage declined in 12 of 21 GMUs by an average of 9% (range = 2–18%). Within these GMUs, high-energy species declined by an average of 15% (range = 10–26%). Predicted changes in forage quantity were driven by the change over time in canopy cover and time since fire. Increases in total and high-energy forage in other GMUs ranged from 2 to 16% and 4 to 21%, respectively. The largest predicted increases in forage occurred in areas where forest fires had occurred in the 2000s (GMUs 17, 19, and 20; Fig 3B).

### Diet quality

Forage species consumed by moose in northern Idaho varied markedly in energy content, and only half of them met estimated energetic demands of a non-lactating, non-pregnant female moose during summer (10.9 kJ/g DE; S1 Text). Overall, mean DE values for leaves (10.9 kJ/g dry matter, range = 9.6–12.9 kJ/g) were about 50% higher than for stems (7.0 kJ/g dry matter, range = 5.1–9.5 kJ/g). Because the summer diets of moose consist primarily of leaves, we evaluated diet quality relative to summer energetic costs based on DE for leaves only. Fifty percent of forage species were below the estimated DE threshold of 10.9 kJ/g dry matter needed to meet daily energetic requirements during summer (Table 1). Species consumed by moose that were categorized as high-energy forage (DE >11.3 kJ/g) were redosier dogwood, Pacific yew, evergreen ceanothus, and redstem ceanothus. Moderate-energy forage species (11.3 kJ/g > DE > 10.5 kJ/g) were bitter cherry, mallow ninebark, and thimbleberry. Forage species categorized as low energy (DE <10.5 kJ/g) were alder-birch spp., willow spp., honeysuckle, common snowberry, and huckleberry spp.

Like energy, protein content differed among forage species consumed by moose in our study area, and DP of stems was lower than that of leaves. Mean DP of leaves (5.49 g/100g, range = 1.73–7.90) was about 300% higher than stems (1.35 g/100g, range = 0–3.72) on a dry matter basis (Table 1). Nevertheless, the leaf DP of roughly 1/3 of forage species failed to offset estimated daily MFN and EUN losses (4.3 g/100g; S1 Text). Species that were categorized as high-protein forage (DP >6.5 g/100g) were ceanothus spp., bitter cherry, and alder-birch spp.; moderate-protein species (6.5 g/100g > DP ≥ 4.3 g/100g) were redosier dogwood, willow spp., thimbleberry and snowberry; mallow ninebark, honeysuckle, huckleberry, and Pacific yew were categorized as low-protein forage (DP <4.3 g/100g; Table 1). The relative ranking of forage shrubs based on DP differed slightly from DE in that alder-birch spp. and willow spp. were ranked higher based on protein content than energy. Ceanothus spp. were the only forage shrubs ranked as high based on both energy and protein content (Table 1).

### Correlations between population trends and forage parameters

**Fig 4:**
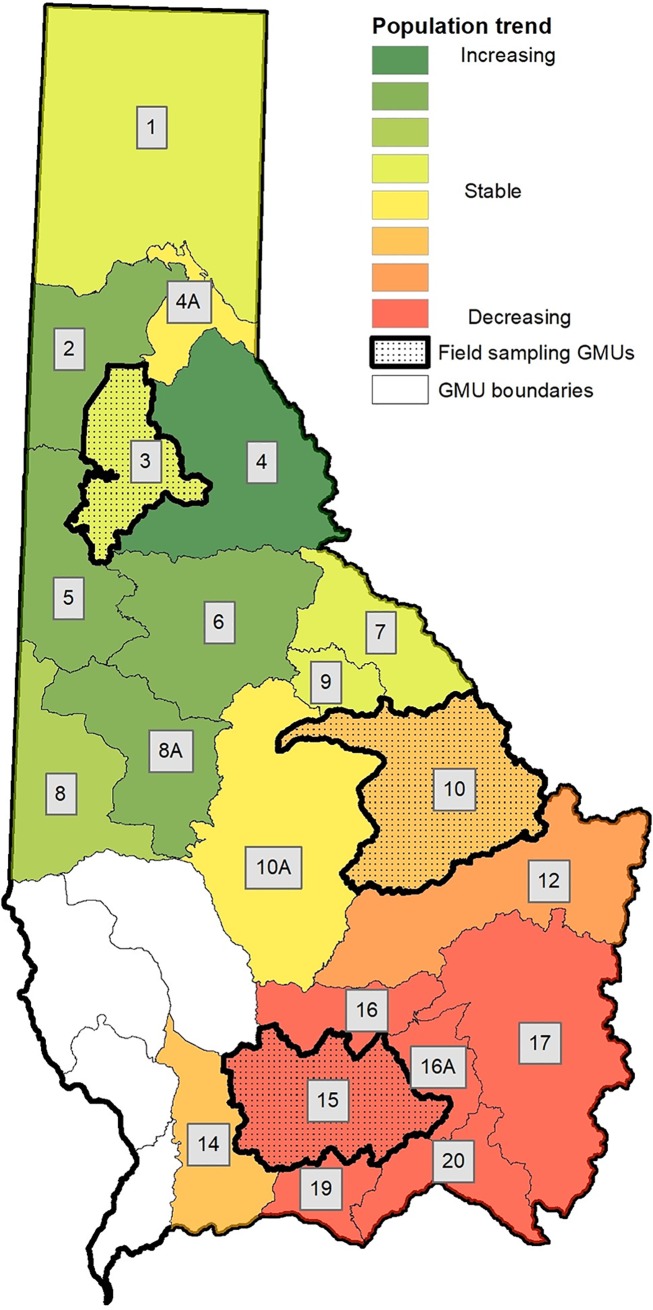
Trends in moose population index. Spatial distribution of a qualitative index of moose population trends estimated from harvest and management data since 1984 for 21 Game Management Units (GMUs) in northern Idaho, USA. Zero values were classified as ‘stable’ and positive and negative values indicate an increasing or decreasing trend, respectively.

The index of population trend varied among moose populations across northern Idaho. Most populations in the northern half of the study area were estimated to be increasing, whereas the strongest declines occurred in the southern portion of the study area (Fig 4). Only 2 GMUs (GMUs 4A and 10A) were estimated to have stable populations. We excluded three GMUs (GMUs 17, 19, and 20) from analyses of correlation with predicted forage because forage estimates in those units were driven by recent large forest fires, and the population trend index prior to 2013 would not be sensitive to such recent disturbances. In addition, these GMUs were closed to hunting in 2013, precluding incorporation of recent harvest data.

The index of population trend was positively associated with predicted values for both the current quantities of forage shrubs and the percent change in quantity of forage since 1984. Population trend was most strongly correlated with the percent change in quantity of moderate-energy forage (*r* = 0.60), but current total forage and current moderate-energy forage also were strongly correlated with population trend (Table 3, S1 Fig). Current quantities of moderate and low-protein forage shrubs also were positively associated with the population trend index. Overall, the qualitative index of population trend was correlated with forage parameters, and moose populations estimated to be increasing were generally associated with higher quantities of forage shrubs. Likewise, GMUs that had predicted declines in forage quantity tended to have declining population trends in contrast to GMUs with predicted increases in available forage.

**Table 3:** Correlations (Pearson correlation coefficient, *r*) between an index of moose population trend and estimates of current forage volume (cm^3^/m^2^) and percent change in forage volume (1984–2016) for 18 game management units in northern Idaho, USA. Forage Shrubs are Grouped by Relative Measures of Forage Quality (Protein and Energy).

Quantity of forage (cm^3^/m^2^)	Population trend index	p-value
Total forage	0.54	0.019
High-energy forage	0.43	0.072
Moderate-energy forage	0.52	0.029
Low-energy forage	0.07	0.778
High-protein forage	0.29	0.240
Moderate-protein forage	0.56	0.015
Low-protein forage	0.48	0.044
% Change in total forage	0.23	0.358
% Change in high-energy forage	0.35	0.153
% Change in moderate-energy forage	0.60	0.009
% Change in low-energy forage	0.14	0.593
% Change in high-protein forage	0.30	0.227
% Change in moderate-protein forage	0.10	0.707
% Change in low-protein forage	-0.01	0.975

## Discussion

Documented declines in disturbance due to a reduction in timber harvest and fire suppression [3,62] have resulted in advancing forest succession within the study area, which has the potential to alter the foraging landscape for moose. We quantified variation in quantity and quality of moose forage in response to changes in forest structure across a broad spatial extent (36,654 km^2^). Furthermore, we illustrated how availability and changes in relative availability of forage shrubs might be linked to moose population trends. Our results indicate that forage conditions might be influencing moose populations in northern Idaho. Traditional methods for estimating forage biomass, such as double sampling techniques [46], are impractical to implement at broad extents because they are time and labor intensive [63]. By integrating established field sampling methods with recent advances in remote sensing analyses in a modeling framework, we created spatially explicit predictions of current and past quantity of forage shrubs across northern Idaho. Our application of this approach indicated that advancing forest succession across the study area over 3 decades has reduced availability of important summer forage shrubs for moose.

The primary drivers of predicted changes in forage were time since fire and percent tree cover. Counterintuitively, several shrub species had a positive relationship with tree cover (S5 Table). This is likely due in part to delayed recruitment and growth of shrubs following disturbance. Shade tolerance also may explain this association; shrub species can persist below some species specific threshold level of tree cover, and the effect of this threshold can be seen in the quadratic term for tree cover in our models. Coefficients for that term were either negative, indicating a decline in the probability of presence above a species specific cover threshold, or zero, indicating no effect for highly shade tolerant shrubs. It also is important to consider the relative importance of each forage species to moose when interpreting model coefficients. Forage species likely to be important include those that have high volume (Table 2) that also have a high percent occurrence within the individual diets, and/or have a high mean dietary proportion, and/or are of higher nutritional quality (Table 1). These species include willow spp., mallow ninebark, bitter cherry, alder-birch spp., and ceanothus spp., whereas other species like huckleberry spp., thimbleberry, and snowberry are likely less important because they are relatively uncommon among the diets, are consumed in lesser amounts, and contribute less overall biomass to total forage (Table 2). Two fire adapted forage species likely to be important exhibited a negative relationship with time since fire, ceanothus spp., which require fire to germinate [64] and willow spp., which have higher recruitment and survival on burned sites [65]. The predicted decline of these species in the absence of fire is likely to have a greater effect on moose that is not offset by a predicted increase in less important forage species like honeysuckle, huckleberry, and snowberry, which are more shade tolerant, not fire dependent, and have low shrub volume (Table 2). Despite positive relationships between tree cover and time since fire for some forage species, overall forage quantity was predicted to have declined and a qualitative index of moose population change was correlated with these changes, indicating that variation in availability of forage shrubs across space and time could be affecting moose population dynamics.

Moose are the largest browser in North America, and therefore they require a greater absolute volume of forage compared to smaller browsers such as mule deer. Consequently, moose likely face a tradeoff between forage quality and abundance. A review of moose foraging ecology by [36] showed that moose tend to balance forage quality with increased quantity to maximize DE intake. Therefore, availability of forage, even of moderate-quality species, is likely an important driver of foraging behavior of moose in northern Idaho. Indeed, similar patterns of behavior have been documented for moose in other studies [66]. Moose often select forage species that allow them to crop large bites, so they can maintain sufficiently high harvesting rates to meet daily nutrient requirements [36]. Therefore, forage species that offer small bites such as common snowberry, huckleberry, grasses, and forbs are likely to be used less when shrubs that allow more efficient foraging are available.

Because of their large body size, forage consumption by moose during summer also might be influenced by thermal constraints on habitat use that result in greater consumption of shade-tolerant species or lower-quality shrubs. Our study area is near the southern boundary of the distribution of moose in North America, and there is growing evidence that warm temperatures influence habitat selection by moose during summer [67–69]. Ceanothus spp. were high in both protein and energy; however, based on field observations and our change detection analysis, ceanothus spp. also were among the least shade-tolerant shrubs that occurred in the diets of moose in our study area. Thus, if moose commonly select closed-canopy forests for thermoregulatory reasons, such shrubs might not be readily available.

Predicted quantities of forage shrubs varied considerably across the study area and were generally higher for northern GMUs that were dominated by western red cedar and western hemlock PNV series (Fig 3A). These vegetation types are associated with high levels of soil moisture and include the two most productive series for timber in northern Idaho [42]. Canopy cover, as an index to forest successional stage, did not explain all of the variation in predicted availability of forage shrubs among GMUs, indicating that environmental variables that determine PNV also contribute to shrub productivity. GMUs predicted to have lower amounts of forage were dominated by grand fir or subalpine fir PNV series. The grand fir series occurs at drier sites, and the subalpine fir series is dominated by shade-tolerant species that tend to be associated with colder, less productive sites [42]. The relationship between shrub productivity and PNV likely applies to other areas and could be used as a first approximation to potential availability of forage shrubs for moose in addition to assessing forest successional stage.

Predicted changes in forage quantity since 1984 also varied considerably among GMUs (Fig 3B), with declines in forage predicted across the study area. A growing recognition of the non-utilitarian values of national forests has resulted in reduced timber harvest, and we predicted that these conditions, along with fire suppression, would result in reduced forage availability on national forest lands. Indeed, 10 of 12 GMUs that were predicted to have declines in forage consisted predominately of national forest lands. Canopy cover, as an index to forest successional stage, in addition to the covariate time since fire, were the primary drivers of forage quantity change. Out of a total of 9 GMUs that were predicted to have increases in forage, 4 consisted entirely of national forest lands that experienced forest fires and the remaining 5 consisted of extensive private and Idaho state lands where timber harvest was a predominant source of disturbance. Additionally, GMUs where forage was predicted to have declined the most were associated with western red cedar and western hemlock PNV series, which might be due to the high productivity of these habitat types facilitating more rapid forest succession, increased canopy closure, and reduced abundance of forage shrubs [70–71]. These results highlight how both fire and timber harvest can be used to create and maintain early seral forests to benefit moose, while also identifying GMUs where these tools can be put to effectual use.

Individual forage species respond differently to disturbance and therefore it is important to consider the ecology of these forage species when managing them across a landscape. Ceanothus species were the most sensitive to increases in canopy cover necessitating frequent disturbance to maintain them on the landscape. Low to moderate-intensity burns improve redstem ceanothus establishment and growth [72] while moderate to high intensity burns are a necessity for germinating evergreen ceanothus seeds [64]. Redstem ceanothus remains abundant when burned every 10 to 15 years, but vigor and abundance degenerates quickly without periodic fires [72]. Ceanothus spp. also are nitrogen fixers [73] and consequently might be important in maintaining soil fertility. Shrubs of moderate-energy that were consumed often in our study included mallow ninebark and bitter cherry. These species do not appear to require frequent management attention as they are highly available and more shade tolerant than ceanothus spp, however, bitter cherry can quickly grow beyond browsing reach after several years [72]. Willow spp. (predominately Scouler willow) has higher recruitment and survival on heavily burned sites [65]. High severity fires that kill live foliage result in vigorous sprouting from the root crown [72], however, willows also can colonize disturbed areas via windborne seeds [74]. Finally, although alder-birch spp. generally respond well to fire and logging activities, they also can persist into late successional stages and are frequently found in moist riparian areas such as along streams, wet meadows, and seeps [42]. In summary, most summer moose forage species in northern Idaho are fire adapted and, except for alder-birch spp., are relatively shade intolerant.

We made several simplifying assumptions when modeling forage quantity across the landscape. First, because we could not quantify potential variation in size of shrubs across the study area, we used mean measured values of shrub volume together with predictions of shrub presence to estimate volume of potential forage. Inclusion of variation in shrub size would likely improve estimates of forage volume and contribute to greater variation in forage quantity estimates among GMUs. Variation in shrub volume across the landscape is likely influenced by many factors, such as past fire intensity and variation in logging practices, which are not easily captured with remotely sensed covariates. In addition, some biologically relevant remotely sensed covariates might not be sufficiently accurate at the scales needed to model variation in shrub size. One potential path forward might be to use lidar data to directly measure shrub volume rather than attempt to model volume. In this study, variation in shrub size is probably a minor factor relative to accurately predicting occurrence of shrub species within the relatively fine-scale Ecognition polygons and comparing them at the much broader spatial scale of GMUs. Nonetheless, estimated differences among GMUs should be interpreted as relative differences. Second, predicted forage values for all 21 GMUs were derived from shrub presence models constructed with field data from 3 GMUs. The 3 sampled GMUs were selected to represent the range of environmental variation across the northern Idaho study area. Increased uncertainty is unavoidable, however, when extrapolating beyond the sampled areas, and thus our results should be interpreted with caution. Third, although browsing by moose can alter forest regeneration at stand and regional scales [75–76], we did not incorporate estimates of moose density or browsing intensity into our assessment of change over time, in part because solid data on those parameters were not available. Fourth, although we conducted cross-validation to assess model fit, we did not collect additional field data to validate model predictions. Finally, in our exploration of the relationship between population trends and predicted forage quantity and change in quantity, we grouped shrubs by relative forage quality measures (i.e., energy and protein). Other metrics for categorizing shrubs (e.g., based on diet composition or forage selection) might be useful in helping to explain population responses. Nonetheless, predicted forage quantity and change in forage over time were correlated with a qualitative index of population trend, indicating that at a coarse resolution, availability of forage could be a meaningful driver of variation in moose populations across northern Idaho.

Although moose populations are declining across much of their range in North America, recent (i.e., 1990s) high numbers of moose in our study region were likely not representative of historical norms. Few observations of moose in northern Idaho were recorded before 1900, and early explorers in the area did not record moose in the 1800s [77]. The expansion of moose populations into northern Idaho began in the 1950s and was likely facilitated by increasing timber harvest and large forest fires. For example, forest fires burned over 60% of the study area between 1910 and 1960; in contrast, only about 12% of the study area burned between 1961 and 2000 [78]. Moose largely rely on early seral habitat, and there are many examples of range expansion [79] and population increases [80–81] associated with forest fires and logging. Other population drivers that warrant further consideration that were not evaluated in this study include predation pressure, climate related impacts, and disease. Black bears, mountain lions, and wolves are present throughout the study area at varying densities and little is known about predation rates on moose or the extent to which predation could be a primary or proximate cause of decline [27–28]; however, anecdotal evidence suggests some moose populations began to decline prior to the establishment of wolves (IDFG). A warming climate also has potential to influence moose populations through several mechanisms including food-cover tradeoffs [67], and direct influences on plant phenology and spring green up [32]. Finally, little is known about the prevalence or impact of various parasites and diseases found in other studies such as winter ticks [82], brain worm [29], tapeworms [28] and arterial worm [83]. It is likely that a suite of interacting factors including forage quality are driving population trends, however, without continued habitat disturbances that create early seral vegetation communities, moose populations are likely to decline.

## Conclusions

Our results indicate that variation in quantity of forage for moose across northern Idaho is likely correlated with moose population trends. These correlations indicate that forage might play an important role in limiting moose populations and contributing to population declines in our study region. Research linking forage conditions with foraging behavior, nutritional condition and fitness of individuals is needed to elucidate the mechanisms underpinning these relationships [12]. We recommend that future research on moose populations include consideration of the foraging landscape and its potential interaction with other population drivers. Failing to do so could result in misidentification of proximate population drivers as ultimate factors, and consequently, management actions that do not produce expected results. In addition, the relative importance of different population drivers, including forage limitation, predation, or climatic factors, is likely to change across time and space, stressing the need for data collection across large spatial scales and long timeframes. Our study highlights the importance of assessing how changes in land management across broad spatiotemporal extents affect wildlife and their habitats. This knowledge can be used in forest management to assess and prescribe disturbances (e.g., timber harvest and forest fires) required to maintain productive wildlife populations.

## Supporting information

S1 Table Biophysical characteristics of Game Management Units. Data exclude areas of non-moose habitat (e.g., urban areas and agriculture).
(DOCX)

S2 Table Environmental covariates used to model shrub presence and volume. (DOCX)

S3 Table Criteria used to assign trend index values for moose populations. Data are based on harvest from 1984 to 2016 for each game management unit (GMU) in northern Idaho, USA. An overall population trend index was calculated by summing the assigned values for each data source.
(DOCX)

S4 Table Summary of diet results. Percent occurrence and mean dietary proportions of forage species detected using microhistological analyses of 43 fecal samples collected in northern Idaho, USA. Shrub species with mean dietary proportions <3% (bolded) were excluded from analyses.
(DOCX)

S5 Table Mean coefficient values for environmental covariates used to predict shrub presence. Mean values were generated by cross-validation of the lasso regression model repeated 30 times to reduce variability in the results due to random allocation of the observations to sub-samples used for cross-validation. Coefficients with a value of zero were effectively dropped from the model by the cross-validated regularization of the lasso regression model. Lasso regression tends to "shrink" parameters to zero for covariates that do not contribute to the predictive accuracy of the model. Covariate abbreviations described in footnote.
(DOCX)

S1 Fig Correlations between an index of moose population trend and estimates of forage volume. (DOCX)

S1 Text Detailed methods for supporting analyses. (DOCX)
