# Supplementary material for: Linking forest management to moose population trends: The role of the nutritional landscape
Source: PLoS One. 2019 Jul 16;14(7):e0219128. doi: 10.1371/journal.pone.0219128 (PMC6634377; doi:10.1371/journal.pone.0219128)
Supplement: S1 Table — Data exclude areas of non-moose habitat (e.g., urban areas and agriculture). (DOCX) [file pone.0219128.s001.docx]

**S1 Table.** **Biophysical characteristics of Game Management Units.** Data exclude areas of non-moose habitat (e.g., urban areas and agriculture).

| GMU | Dominant Potential Natural Vegetation (PNV) types^a^ | Area (km^2^) | Mean min./max. temp. (°C) | Mean annual precip. (cm)^3^ | Elevation range (m) | Percent area with canopy cover 0 - 33% | Percent area with canopy cover 34 - 66% | Percent area with canopy cover 67 - 100% | USFS Forest Service (Percent National Forest) | State of Idaho (Percent State Land) | Percent Private Land |
| --- | --- | --- | --- | --- | --- | --- | --- | --- | --- | --- | --- |
| 1 | TSHE/ABLA | 5,200 | -10 / 28 | 1,030 | 530 to 2,345 | 16% | 32% | 52% | 59% | 19% | 22% |
| 2 | THPL | 1,000 | -7 / 28 | 840 | 625 to 1,550 | 35% | 37% | 28% | 10% | 16% | 74% |
| 3 | TSHE/ABGR | 1,110 | -7 / 29 | 995 | 650 to 1,725 | 16% | 27% | 57% | 63% | 1% | 36% |
| 4 | TSHE | 3,075 | -8 / 28 | 1,150 | 650 to 2,065 | 11% | 26% | 63% | 70% | 4% | 26% |
| 4A | TSHE/ABGR | 400 | -7 / 28 | 940 | 630 to 1,940 | 9% | 25% | 66% | 87% | 0% | 13% |
| 5 | TSHE/THPL | 900 | -6 / 29 | 805 | 650 to 1,500 | 44% | 37% | 18% | 1% | 4% | 95% |
| 6 | TSHE/THPL | 2,630 | -9 / 29 | 1,160 | 650 to 2,075 | 27% | 27% | 45% | 42% | 9% | 49% |
| 7 | THPL/TSME | 1,420 | -10 / 28 | 1,240 | 755 to 2,300 | 14% | 41% | 45% | 88% | 0% | 12% |
| 8 | THPL | 295 | -6 / 29 | 830 | 780 to 1,510 | 40% | 37% | 23% | 3% | 14% | 83% |
| 8A | THPL | 1480 | -7 / 31 | 930 | 395 to 1,690 | 29% | 25% | 46% | 33% | 15% | 52% |
| 9 | THPL/TSME | 510 | -9 / 28 | 1,260 | 695 to 2,125 | 12% | 35% | 53% | 79% | 0% | 21% |
| 10 | THPL/TSME/ABLA | 2,980 | -11 / 30 | 1,395 | 490 to 2,300 | 18% | 39% | 43% | 98% | 2% | 0% |
| 10A | THPL | 3,665 | -8 / 32 | 1,070 | 300 to 1,930 | 26% | 26% | 48% | 25% | 26% | 49% |
| 12 | ABLA/ABGR | 2,830 | -12 / 31 | 1,230 | 435 to 2,580 | 20% | 40% | 40% | 94% | 0% | 6% |
| 14 | ABGR/ABLA | 940 | -12 / 32 | 905 | 520 to 2,445 | 27% | 45% | 28% | 83% | 6% | 11% |
| 15 | ABGR/ABLA | 2,120 | -12 / 31 | 865 | 470 to 2,435 | 10% | 32% | 58% | 92% | 0% | 8% |
| 16 | THPL | 850 | -10 / 32 | 975 | 390 to 2,245 | 14% | 26% | 60% | 89% | 2% | 9% |
| 16A | ABGR/ABLA | 690 | -12 / 31 | 990 | 525 to 2,315 | 16% | 41% | 42% | 100% | 0% | 0% |
| 17 | ABGR/ABLA/PSME | 3,400 | -13 / 31 | 1,050 | 530 to 2,845 | 37% | 42% | 21% | 100% | 0% | 0% |
| 19 | ABGR/ABLA | 400 | -12 / 29 | 850 | 875 to 2,570 | 42% | 47% | 11% | 100% | 0% | 0% |
| 20 | ABGR/ABLA | 915 | -13 / 29 | 940 | 980 to 2,680 | 32% | 46% | 22% | 100% | 0% | 0% |
| ^a^ABGR-grand fir; ABLA-subalpine fir; THPL-western red cedar; TSHE-western hemlock; TSME-mountain hemlock. | | | | | | | | | |  |  |
