# Supplementary material for: Linking forest management to moose population trends: The role of the nutritional landscape
Source: PLoS One. 2019 Jul 16;14(7):e0219128. doi: 10.1371/journal.pone.0219128 (PMC6634377; doi:10.1371/journal.pone.0219128)
Supplement: S2 Table — (DOCX) [file pone.0219128.s002.docx]

**S2 Table.** **Environmental covariates used to model shrub presence and volume.**

| Covariate | Description |
| --- | --- |
| Elevation | Digital elevation model |
| Topographic wetness index | Steady-state wetness index |
| Topographic position index | Describes elevation of cell relative to elevation of specified neighborhood |
| Solar radiation | Estimated solar radiation of cell |
| Sine and cosine of aspect | Decomposes aspect into north-south and east-west components |
| Heat load index | Estimates direct radiation from latitude, slope, and aspect |
| Available water supply | Soil water storage capacity ^a^ |
| Soil percent clay | Soil percent clay^a^ |
| Soil percent sand | Soil percent sand^a^ |
| Soil percent silt | Soil percent silt^a^ |
| Soil organic matter | Soil organic matter^a^ |
| Soil cation-exchange capacity | Soil cation-exchange capacity (measure of nutrient content)^a^ |
| Soil depth to restrictive layer | Soil depth to any restrictive layer (cm) |
| Soil pH | Soil pH^a^ |
| 30-year average min precip | Downscaled from 30-year normals (1981 to 2010) from PRISM Climate Group |
| 30-year average max precip | Downscaled from 30-year normals (1981 to 2010) from PRISM Climate Group |
| 30-year average annual precip | Downscaled from 30-year normals (1981 to 2010) from PRISM Climate Group |
| 30-year average min temp | Downscaled from 30-year normals (1981 to 2010) from PRISM Climate Group |
| 30-year average max temp | Downscaled from 30-year normals (1981 to 2010) from PRISM Climate Group |
| Percent tree cover | Percent tree cover  (Nlcd.gov) |
| Time since fire | Number of years since last fire  (Fs.usda.gov) |

^a^Soil parameters measured in the 0-25 cm depth layer.
