# Supplementary material for: Linking forest management to moose population trends: The role of the nutritional landscape
Source: PLoS One. 2019 Jul 16;14(7):e0219128. doi: 10.1371/journal.pone.0219128 (PMC6634377; doi:10.1371/journal.pone.0219128)
Supplement: S3 Table — Data are based on harvest from 1984 to 2016 for each game management unit (GMU) in northern Idaho, USA. An overall population trend index was calculated by summing the assigned values for each data source. (DOCX) [file pone.0219128.s003.docx]

**S3 Table.** **Criteria used to assign trend index values for moose populations**. Data are based on harvest from 1984 to 2016 for each game management unit (GMU) in northern Idaho, USA. An overall population trend index was calculated by summing the assigned values for each data source.

| Data Source | Criteria | | | | |
| --- | --- | --- | --- | --- | --- |
| Harvest success rate | Sustained between 80 and 100% | Increased by 20 to 39 percentage points | Increased or decreased by < 20 percentage points | Declined by 20 to 39 percentage points | Declined by ≥ 40 percentage points |
| Assigned value | 2 | 1 | 0 | -1 | -2 |
| No. of permits offered | Increased ≥ 300% | Increased ≥ 100% but < 300% | Increased or decreased by < 100% | Increased and then declined to within 25% of historical lows | Declined below historical levels by > 25% or harvest season closed |
| Assigned value | 2 | 1 | 0 | -1 | -2 |
| No. of days hunted | Decreasing trend | Stable trend | Increasing trend or harvest season closed |  |  |
| Assigned value | 1 | 0 | -1 |  |  |
