# Supplementary material for: Linking forest management to moose population trends: The role of the nutritional landscape
Source: PLoS One. 2019 Jul 16;14(7):e0219128. doi: 10.1371/journal.pone.0219128 (PMC6634377; doi:10.1371/journal.pone.0219128)
Supplement: S4 Table — Percent occurrence and mean dietary proportions of forage species detected using microhistological analyses of 43 fecal samples collected in northern Idaho, USA. Shrub species with mean dietary proportions <3% (bolded) were excluded from analyses. (DOCX) [file pone.0219128.s004.docx]

**S4 Table. Summary of diet results.** Percent occurrence and mean dietary proportions of forage species detected using microhistological analyses of 43 fecal samples collected in northern Idaho, USA. Shrub species with mean dietary proportions <3% (bolded) were excluded from analyses.

| Vegetative classes | Percent of Diets | Mean Dietary Proportion |
| --- | --- | --- |
| Shrubs | 100% | 59% |
| Conifers | 88% | 15% |
| Grasses | 57% | 18% |
| Forbs | 21% | 7% |
|  |  |  |
|  |  |  |
| Shrub Species | Percent of Diets | Mean Dietary Proportion |
| Willow spp. (*Salix spp.*) | 88% | 14% |
| Mallow ninebark (*Physocarpus malvaceus*) | 63% | 17% |
| Bitter cherry (*Prunus emarginata*) | 60% | 13% |
| Alder-birch spp. (*Alnus* & *Betula spp.*) | 49% | 14% |
| Ceanothus spp. (*Ceanothus*) | 33% | 24% |
| Honeysuckle spp. (*Lonicera*) | 33% | 10% |
| Redosier dogwood (*Cornus stolonifera*) | 19% | 12% |
| Common snowberry (*Symphoricarpus albus*) | 16% | 7% |
| Huckleberry spp. (*Vaccinium spp.*) | 16% | 8% |
| Thimbleberry (*Rubus parviflorus*) | 14% | 6% |
| Pacific yew (*Taxus brevifolia*) | 12% | 24% |
| **Labrador tea (*Ledum groenlandicum*)** | 16% | 2% |
| **Rocky Mountain maple (*Acer glabrum*)** | 7% | 1% |
| **Oregon grape spp. (*Mohonia spp.*)** | 7% | 1% |
| **Black hawthorn (*Crataegus douglasii*)** | 5% | 1% |
| **Ocean spray (*Holodiscus discolor*)** | 5% | 2% |
| **Elderberry spp. (*Sambucus spp.*)** | 2% | 2% |
|  |  |  |
| Tree Species | Percent of Diets | Mean Dietary Proportion |
| Pine spp. (*Pinus spp.*) | 71% | 9% |
| Douglas fir (*Pseudostuga menziesii*) | 29% | 2% |
| Engelmann spruce (*Picea engelmannii*) | 21% | 9% |
| Western red cedar (*Thuja plicata*) | 17% | 13% |
| Fir spp. (*Abies spp.*) | 12% | 3% |
| Hemlock spp. (*Tsuga spp.*) | 7% | 29% |
