# Supplementary material for: Linking forest management to moose population trends: The role of the nutritional landscape
Source: PLoS One. 2019 Jul 16;14(7):e0219128. doi: 10.1371/journal.pone.0219128 (PMC6634377; doi:10.1371/journal.pone.0219128)
Supplement: S5 Table — Mean values were generated by cross-validation of the lasso regression model repeated 30 times to reduce variability in the results due to random allocation of the observations to sub-samples used for cross-validation. Coefficients with a value of zero were effectively dropped from the model by the cross-validated regularization of the lasso regression model. Lasso regression tends to "shrink" parameters to zero for covariates that do not contribute to the predictive accuracy of the model. Covariate abbreviations described in footnote. (DOCX) [file pone.0219128.s005.docx]

**S5 Table.** **Mean coefficient values for environmental covariates used to predict shrub presence**. Mean values were generated by cross-validation of the lasso regression model repeated 30 times to reduce variability in the results due to random allocation of the observations to sub-samples used for cross-validation. Coefficients with a value of zero were effectively dropped from the model by the cross-validated regularization of the lasso regression model. Lasso regression tends to "shrink" parameters to zero for covariates that do not contribute to the predictive accuracy of the model. Covariate abbreviations described in footnote.

| Forage species | Intercept | Elev | Sin | Cos | TWI | TWI^2^ | TPI | TPI^2^ | HLI | HLI^2^ |
| --- | --- | --- | --- | --- | --- | --- | --- | --- | --- | --- |
| Willow spp. | -12.7063 | 0.0067 | 0.0049 | -0.0281 | 0.0193 | -0.0127 | -0.3551 | 0.2525 | -4.3891 | 0.0000 |
| Mallow ninebark | 4.5263 | -0.0006 | -0.0163 | -0.0031 | -0.1248 | -0.0035 | 0.0882 | -0.0002 | 0.0000 | 1.2904 |
| Bitter cherry | -25.6483 | 0.0018 | 0.0341 | -0.0600 | -0.2669 | 0.0027 | 1.0654 | 0.1381 | -44.1783 | 35.6475 |
| Alder-birch spp. | 2.7556 | 0.0000 | 0.0000 | -0.0003 | 0.0195 | 0.0348 | -0.6999 | 0.0000 | -5.8081 | 0.0000 |
| Redstem ceanothus | -38.4386 | 0.0171 | 0.0656 | -0.0026 | 0.2260 | -0.0642 | -0.4830 | -1.4118 | 11.2157 | -1.8093 |
| Evergreen ceanothus | -3.3216 | 0.0012 | 0.0000 | -0.0028 | -0.4326 | -0.0001 | 0.0000 | 0.0000 | 0.0000 | 4.2900 |
| Honeysuckle | -1.6890 | 0.0026 | -0.0010 | -0.1073 | -0.0512 | 0.0025 | -1.6735 | -1.3027 | -0.1245 | -0.6393 |
| Redosier dogwood | -9.1791 | 0.0000 | -0.0011 | -0.0008 | -0.0013 | 0.0000 | -1.9078 | -0.2544 | -2.6762 | 0.0000 |
| Common snowberry | 5.3746 | 0.0000 | -0.0239 | -0.0534 | -0.0111 | 0.0216 | -0.0030 | -0.1830 | 0.0000 | -0.0214 |
| Huckleberry | -7.5457 | 0.0026 | 0.0224 | -0.0450 | -0.0818 | 0.0000 | -0.0829 | 0.0000 | -1.1955 | -0.0693 |
| Thimbleberry | -50.1013 | 0.0080 | 0.0297 | 0.0292 | 0.2800 | -0.0443 | -0.4792 | 0.9887 | -23.3767 | 18.7458 |
| Pacific Yew | -14.9850 | 0.0014 | -0.0012 | -0.0055 | -0.0027 | 0.0028 | -0.7467 | 0.1588 | 0.7122 | -0.6301 |
|  |  |  |  |  |  |  |  |  |  |  |
|  | Sand | Silt | pH | Organic | Depth | Clay | CEC | AWS | AWS^2^ | TC |
| Willow spp. | 0.0000 | 0.0001 | 0.6517 | -0.0254 | -0.0075 | -0.0606 | -0.0011 | 0.3693 | 0.0000 | 0.0667 |
| Mallow ninebark | 0.0000 | 0.0000 | -0.0932 | 0.0017 | -0.0002 | 0.0001 | 0.0018 | 0.0000 | -0.0092 | 0.0001 |
| Bitter cherry | 0.0209 | 0.0000 | -0.2859 | -0.0004 | -0.0008 | -0.0030 | -0.0062 | 0.3809 | -0.0158 | 0.0198 |
| Alder-birch spp. | -0.0055 | 0.0001 | 0.0000 | 0.0002 | -0.0076 | 0.0000 | 0.0103 | 0.0000 | 0.0146 | 0.0038 |
| Redstem ceanothus | 0.0000 | -0.0590 | -0.1161 | -0.0448 | 0.0039 | 0.0450 | 0.0658 | -0.1604 | 0.1132 | 0.0049 |
| Evergreen ceanothus | -0.0038 | 0.0000 | 0.0000 | 0.0000 | -0.0029 | 0.0000 | 0.0000 | 0.0046 | 0.0267 | 0.0000 |
| Honeysuckle | 0.0258 | 0.0000 | 0.5948 | 0.0632 | 0.0101 | -0.0265 | -0.0434 | 0.1269 | -0.0269 | 0.0421 |
| Redosier dogwood | 0.0209 | 0.0000 | 0.0122 | -0.0004 | 0.0000 | -0.0013 | -0.0045 | 0.0000 | 0.0000 | 0.0006 |
| Common snowberry | -0.0387 | 0.0000 | 0.3159 | 0.0554 | 0.0025 | 0.0087 | -0.0297 | 0.0000 | -0.0679 | 0.0127 |
| Huckleberry | 0.0628 | 0.0000 | 0.6700 | -0.0294 | -0.0001 | -0.0180 | -0.0173 | 0.8769 | -0.0001 | 0.0498 |
| Thimbleberry | 0.0105 | -0.0478 | 0.2783 | -0.0046 | -0.0026 | 0.0000 | -0.0240 | 0.8091 | 0.0052 | 0.0874 |
| Pacific Yew | 0.0030 | 0.0079 | -0.0154 | 0.0015 | 0.0001 | -0.0025 | 0.0061 | -0.1317 | 0.0085 | -0.0015 |
|  |  |  |  |  |  |  |  |  |  |  |
|  | TC^2^ | TSF | AP | MXP | MNP | (MNP)^2^ | MNT | (MNT)^2^ | MXT | (MXT)^2^ |
| Willow spp. | -0.0010 | -0.0079 | 0.0013 | 0.0022 | 0.0228 | 0.0000 | 0.1417 | 0.0000 | 0.2663 | 0.0000 |
| Mallow ninebark | 0.0000 | 0.0000 | 0.0000 | 0.0000 | -0.0758 | 0.0000 | 0.3487 | -0.0099 | 0.0000 | 0.0000 |
| Bitter cherry | -0.0005 | 0.0177 | -0.0008 | 0.1222 | 0.0032 | 0.0000 | -1.4687 | -0.2166 | 1.1049 | -0.0055 |
| Alder-birch spp. | 0.0000 | 0.0000 | 0.0000 | 0.0000 | 0.0000 | 0.0000 | -0.0006 | 0.0200 | 0.0000 | -0.0013 |
| Redstem ceanothus | -0.0004 | -0.0188 | 0.0114 | 0.0148 | -0.5494 | 0.0052 | -0.2229 | -0.0571 | 1.6105 | -0.0199 |
| Evergreen ceanothus | -0.0003 | 0.0000 | 0.0000 | 0.0000 | 0.0263 | 0.0000 | 0.0000 | -0.0184 | 0.0000 | -0.0001 |
| Honeysuckle | -0.0002 | 0.0037 | 0.0000 | 0.0091 | -0.0037 | -0.0009 | -0.0746 | -0.0173 | 0.0000 | -0.0053 |
| Redosier dogwood | 0.0000 | 0.0000 | 0.0000 | 0.0000 | 0.0039 | 0.0000 | -0.0562 | 0.0044 | 0.2445 | 0.0001 |
| Common snowberry | 0.0000 | -0.0001 | 0.0001 | 0.0000 | -0.0042 | -0.0008 | 0.0000 | -0.0005 | 0.0000 | -0.0030 |
| Huckleberry | -0.0004 | 0.0034 | 0.0026 | 0.0013 | 0.0290 | 0.0008 | -0.0534 | 0.0000 | -0.1675 | -0.0035 |
| Thimbleberry | -0.0011 | -0.0068 | 0.0021 | 0.0693 | 0.0301 | 0.0001 | -0.0674 | -0.0328 | 4.0360 | -0.0787 |
| Pacific Yew | 0.0002 | 0.0019 | 0.0000 | 0.0019 | 0.0634 | -0.0009 | -0.3289 | -0.0090 | 0.2600 | -0.0032 |
| Covariate abbreviations: Elev-elevation; Sin-sine of aspect; Cos-cosine of aspect; TWI-topographic wetness index; TPI-topographic position index; HLI-heat load index; Sand-soil percent sand; Silt-soil percent silt; pH-soil pH; Organic-soil percent organic matter; Depth-Soil depth to restrictive layer; Clay-soil percent clay; CEC-soil cation-exchange capacity; AWS-available water supply; TC-percent tree cover; TSF-time since fire; AP-30-year average annual precip; MXP-30-year average max precip; MNP-30-year average min precip; MNT-30-year average min temp; MXT-30-year average max temp. | | | | | | | | | | |
