# Supplementary material for: Linking forest management to moose population trends: The role of the nutritional landscape
Source: PLoS One. 2019 Jul 16;14(7):e0219128. doi: 10.1371/journal.pone.0219128 (PMC6634377; doi:10.1371/journal.pone.0219128)

**S1 Figure.** Correlations between an index of moose population trend (y-axis) and **Part 1)** estimates of current forage volume (cm^3^/m^2^), and **Part 2)** Percent Change in Forage Volume (from 1984 to 2016) for 18 Game Management Units in Northern Idaho, USA. Categories of forage shrubs are: a) total forage, b) high-energy forage, c) moderate-energy forage, d) low-energy forage, e) high-protein forage, f) moderate-energy forage, and g) low-energy forage.

**Part 1: Current Forage Volume (2016):**

a)
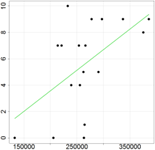
 b)
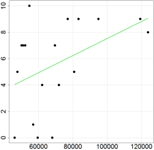
 c)
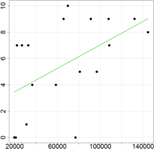
 d)
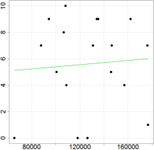
 e)
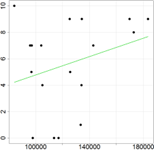


f)
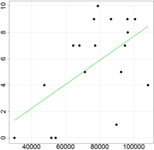
 g)
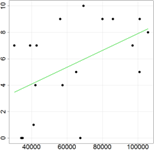


**Part 2: Percent Change in Forage Volume (1984-2016):**

a)
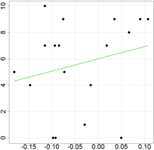
 b)
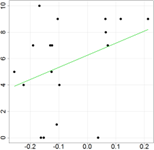
 c)
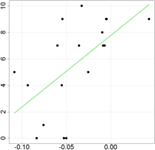
 d)
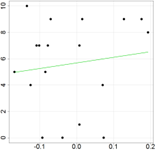
 e)
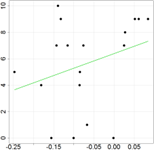


f)
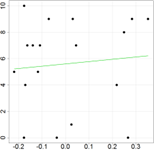
 g)
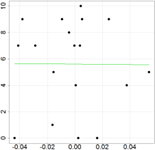

Supplement: S1 Fig — (DOCX) [file pone.0219128.s006.docx]
