# Supplementary material for: Linking forest management to moose population trends: The role of the nutritional landscape
Source: PLoS One. 2019 Jul 16;14(7):e0219128. doi: 10.1371/journal.pone.0219128 (PMC6634377; doi:10.1371/journal.pone.0219128)
Supplement: S1 Text — (DOCX) [file pone.0219128.s007.docx]

**S1 Text. Detailed methods for supporting analyses**.

Estimation of historic canopy cover for models of forage quantity in 1984

Increasing forest canopy closure reduces understory shrub biomass available to wildlife [1]. Because changes in reflectance in the green band were negatively correlated with the amount of tree cover, we used these changes as an index to adjust for changes in canopy cover. We back-calculated tree canopy cover for 1984 by either adding to, or subtracting from, tree canopy cover values from the 2011 National Land Cover Database [2] based on a visual estimate of the magnitude and direction of change in the green band of the Landsat imagery from 1984 to 2011. If little change was observed, no correction was made, but if moderate or large change occurred, canopy cover was either increased or decreased by 35% (moderate change) or 75% (large change). Selection of the 35% and 75% values was based on the rate at which conifer encroachment typically occurs. For example, based on field observations, a 30-year old clearcut with 0% canopy cover has typically regenerated into a relatively closed canopy forest, which we estimated as about a 75% increase in canopy. Threshold values of change in the green band were determined by adjusting each threshold and visually comparing estimated tree canopy cover to the 1984 imagery. The resulting tree canopy cover layer matched with visual estimates of tree cover, and we interpret these estimates to represent relative changes across our study area over time.

To evaluate our 1984 tree cover estimates, we used data from the U.S. Forest Service’s “Ecodata Plot Inventory Database (1940s-1994)”. The data are from plot inventories collected in 1987 and are available at https://www.fs.fed.us/r6/icbemp/. The 1987 was as close as we could find to our 1984 estimates. Each plot includes a point estimates for tree cover. Point estimates can vary tremendously within a small area, and this variation is compounded by location errors. Plot data were screened to remove obviously erroneous data points (e.g., locations that clearly did not match the image – e.g., a rocky outcrop with a high value recorded for tree cover) and to account for disturbance between 1984 and 1987 (e.g., forest fire or timber harvest that occurred between 1984 and 1987). To reduce the effect of location error, we created 90m buffers around each point and calculated the median value for 1984 tree cover within the buffers. Tree cover data from each data source were binned into 10% categories so that the data would be comparable. A total of 43 plots were used from across the study area, and the median 1984 tree cover values are plotted against the plot tree cover below.

The resulting R-squared was 0.68 (a correlation coefficient of 0.83), indicating reasonable agreement between estimates from the two data sources. It is important to note that neither data source can be considered “truth” and comparing satellite-based tree cover estimates to ground-based estimates will increase noise. Another important result is that at tree cover values <80%, our approach tends to overestimate canopy cover for 1984, meaning that the canopy was likely more open than we estimated. More open canopies tend to support more understory growth (moose forage shrubs), and therefore, our predicted declines in forage since 1984 are likely conservative.


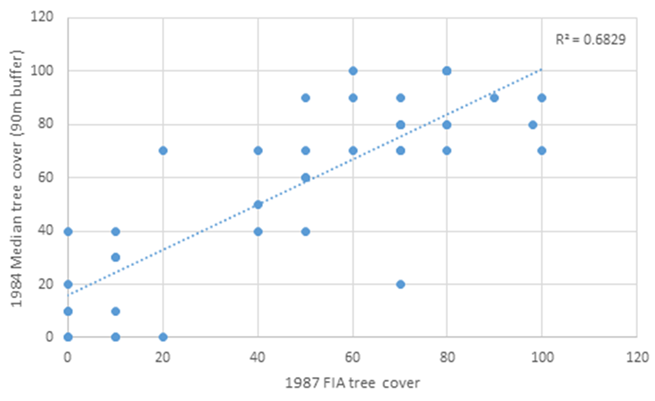


Estimation of nutritional requirements and rankings of diet quality

We categorized shrub species as high, moderate, or low energy based on their estimated DE. A daily energetic cost of 820 kJ/kg BW^0.75^ was estimated for free-ranging, non-lactating, non-pregnant, female moose in Alberta, Canada between July through October [3]. Estimated dry matter forage intake during summer for moose ranges from 116 to 142 g/kg BW^0.75^ per day [4-5]. We used the mean value of dry matter intake (129 g/kg BW^0.75^) to calculate the mean DE in kJ/g needed to meet daily energetic costs. We estimated that 10.9 kJ/g of DE are needed to meet daily energetic costs after correcting for energy lost to urine and methane (18% loss; [6]) and an efficiency coefficient of 71% [7]. Shrubs were then ranked as high, moderate, or low energy if their composited leaf DE was >11.3, between 10.5 and 11.3, or <10.45, respectively. The value of 11.3 was selected because it exceeded maintenance (10.9) and there was a natural break between thimbleberry (11.0) and the next highest DE shrub (redstem ceanothus, 11.6).

We also ranked shrubs according to whether their protein content was sufficient to offset daily metabolic fecal nitrogen (MFN) and endogenous urine nitrogen (EUN) losses for a female moose in summer. We assumed an average body mass of 350 kg (Houston 1969) and daily dry matter intake of 129 g/kg BW^0.75^ [4-5]. Metabolic fecal nitrogen was estimated to be 5.06 g N/kg dry matter intake [8-9], and EUN was estimated to be 56 mg N/kg^0.75^ per day [9]. The DP needed to offset the loss was calculated to be 4.30 g/100g dry matter including an efficiency coefficient of 80% [6]. If the DP value of a shrub was insufficient to offset N loss (i.e., <4.3 g/100g), it was categorized as low in protein. A shrub was categorized as moderate or high in protein if the DP value was between 4.3 and 6.5 g/100g forage, or >6.5 g/100g, respectively.

Literature Cited

1. Peek JM, Korol JJ, Gay D, Hershey T. Overstory-understory biomass changes over a 35-year period in southcentral Oregon. For Ecol Manage. 2001;150: 267-277.
2. Coulston JW, MoisenGG, WilsonBT, FincoMV, Cohen WB, Brewer CK. Modeling percent tree canopy cover: a pilot study. Photogramm Eng Remote Sensing. 2012;78: 715-727.
3. Renecker LA, Hudson RJ. Ecological metabolism of moose in aspen-dominated boreal forests, central Alberta. Can J Zool. 1989;678: 1923-1928.
4. Renecker LA, Hudson RJ. 1985. Estimation of dry matter intake of free-ranging moose. J Wildl Manage. 1985;49: 785-792.
5. Schwartz CC, Renecker LA. Nutrition and energetics. In: Franzmann AW, Schwartz CC, editors. Ecology and management of the North American moose. Washington and London: Smithsonian Institution Press. 1997. Pp. 476–478
6. Robbins CT. Wildlife feeding and nutrition. 2nd ed. San Diego, California: Academic Press; 1993.
7. Hubbert ME. The effect of diet on energy partitioning in moose. Ph.D. Dissertation, University of Alaska, Fairbanks. 1987.
8. Robbins CT, Hanley TA, Hagerman AE, Hjeljord O, Baker DL, Schwartz CC, et al. Role of tannins in defending plants against ruminants: reduction in protein availability. Ecology. 1987;681: 98-107.
9. Schwartz, C. C., W. L. Regelin, and A. W. Franzmann. 1987. Protein digestion in moose. The Journal of Wildlife Management 51:352-357.
